# Falx cerebelli and its associated occipital venous sinus: an anatomical study

**DOI:** 10.1007/s00276-024-03416-8

**Published:** 2024-07-08

**Authors:** Mamatha Hosapatna, Kushi Kunder, Nandini Prashanth Bhat, Ashwija Shetty, Sanjoy Sanyal, Sushma Prabhath, Suhani Sumalatha

**Affiliations:** 1 Department of Anatomy, Kasturba Medical College Manipal, Manipal Academy of Higher Education, Manipal, Karnataka 576104 India; 2 Department of Anatomy, Department Chair of Anatomical Sciences, Richmond Gabriel University College of Medicine, Kingstown, West Indies VC0282 Saint Vincent And The Grenadines

**Keywords:** Falx cerebelli, Occipital sinus, Aberrant sinus, Venous sinus

## Abstract

**Purpose:**

This study presents the morphological variation of falx cerebelli, which helps to identify the possible variations in the presence of the occipital sinus in the posterior margin of the fold whose damage during midline incision of posterior cranial fossa surgeries may lead to internal hemorrhage.

**Method:**

The study was conducted on 48 cranial cavities exploring the falx cerebelli. Variations in the number of folds, its proximal and distal attachments, and the drainage pattern of the occipital sinus were evaluated by histological processing of the upper 1/3rd section of the falx fold.

**Results:**

The variation in the number of folds recorded are single folds in 87.5%, double folded in 8.3%, and multiple folds (five and seven folded) in 4.2% of the cases. The variation in the proximal and distal attachments in single falx folds showed three combinations: Ramified triangular in 66.7%, both ramified type in 12.5%, and both triangular type in 8.3% of the cases. Double and multiple folds showed ramified and triangular types of variation in their attachments. Histological findings showed the presence of occipital venous sinuses in most of the single falx fold. Two aberrant venous sinuses were seen in a double and five-folded falx cerebelli.

**Conclusions:**

This study records the variations in the morphology of falx cerebelli. The histological data of this study sheds light on the drainage pattern of venous sinuses in the area whose negligence during midline incisions of brain surgeries may increase the possibility of hemorrhage.

## Introduction

The human brain and spinal cord are covered by three meningeal layers. They are the outer dura, middle arachnoid, and inner pia maters. The dura mater of the brain has two additional layers, unlike the spinal cord which has only one. They are the outer periosteal layer and the inner meningeal layer. The inner meningeal layer comprises of four types of folds or projections – falx cerebri, falx cerebelli, tentorium cerebelli, and diaphragma sellae [7].

Falx cerebelli is a dural fold projecting in the midline with an apex and base attached to the occipital bone, along the internal occipital crest within the posterior cranial fossa [11].

The occipital sinus is a small dural venous sinus present in the posterior margin of the falx cerebelli attached to the occipital bone [28]. Superiorly, it drains into the confluence of sinuses whereas inferiorly it communicates with the posterior internal vertebral plexus thereby receiving venous flow from the cerebellum and the medulla oblongata and finally drains the fourth ventricle’s choroid plexus [8].

The morphology of falx cerebelli with the presence of a single dural fold is considered normal. However, its variation in the proximal and distal attachment and in some cases presence of duplicated, partially duplicated, triplicated, quadruplet, and five folds of falx cerebelli or complete absence are reported in the medical literature [8, 28].

The variation in the presence of multiple folds of falx cerebelli resulted in the multiplication of occipital sinuses in most documented cases. The falx cerebelli variations can impact the occipital sinuses [7] drainage pattern, leading to the formation of aberrant or unusual drainage conduits [3]. While variations in the falx cerebelli leading to multiple folds seem to be a small anatomical difference, they can have important consequences for the adjacent venous sinuses. Awareness of such variations is a must in the field of neuroscience. Several pathological conditions, such as the herniation of the brain caused by intracranial hemorrhages, intracranial tumors, hydrocephalus, or brain edema, might be fatal due to increased weight on the dural folds, resulting in damage to the folds and the sinus underneath [3, 19]. Thus, the present study aims to identify the morphological variations of falx cerebelli and its associated venous sinuses. So whenever surgical and diagnostic approaches to treat pathological conditions are made, such studies will provide the necessary research data to support them.

## Materials and methods

### Materials

A dissection kit with blunt forceps, toothed forceps, pointed forceps, a scalpel, and scissors is used wherever necessary for neat, scientific, and systematic dissection of the cranial cavity.

### Methods

**Fig. 1:**
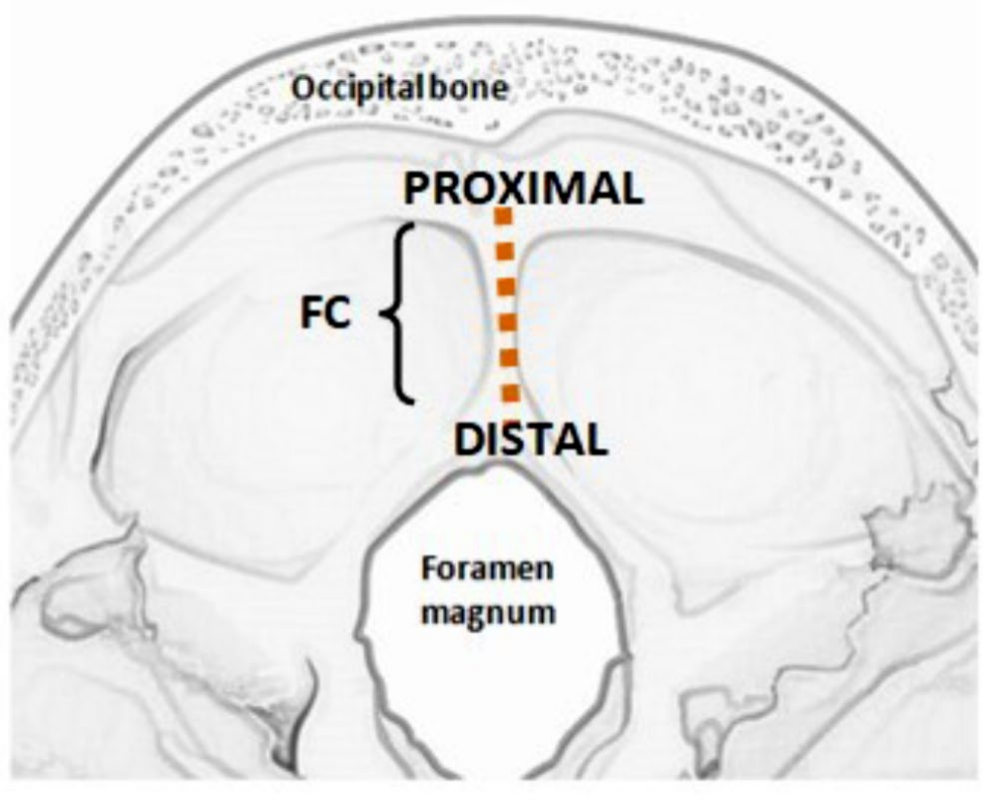
Line diagram showing the attachment of falx cerebelli; FC-Falx cerebelli along the internal occipital crest; PROXIMAL-proximal attachment; DISTAL-distal attachment

A 10% formalin embalmed 48 adult cadavers were taken from the Department of Anatomy, KMC Manipal. Dissection involved the removal of the skull cap followed by carefully opening the dura mater. After dissecting the brain from the brainstem, the tentorium cerebelli was lifted from within the cranial cavity. A sickle-shaped fold in the midline, along the internal occipital crest below the tentorium cerebelli was identified as falx cerebelli. Further following morphological parameters of the falx cerebelli were noted:

- Variations in the number of folds of falx cerebelli were observed and noted - as double, triple, or multiple folds. Further, a small piece of falx was harvested from its midpoint and processed for histological examination to confirm the presence or absence of venous sinus.
- Variations in the shape of the proximal attachment and distal attachment of the falx cerebelli were identified and noted (Fig. 1).

The related contents of the cranial cavity were pinned and photographed. A constant distance between the dissected area and the camera level was always maintained.

### Histological evaluation

This was performed to confirm the association of the occipital sinus/aberrant venous sinus in the folds of the falx cerebelli. Selected tissue of the falx cerebelli was kept for fixation in 10% formalin, followed by dehydration and embedding. After microtomy (5 μm), the mounted slide was subjected to a staining process (H&E stain). Histological specimens were examined and photographed using a microscope, an Olympus model CX 41, and a U-TV1X-2 camera from Tokyo, Japan.

## Results

In the present study, the classical representation of the falx cerebelli, i.e., – single fold was seen in 87.5%, double folds of falx cerebelli were seen in 8.3% and multi-folded falx cerebelli in 4.2% of the specimens.(See table 1).

**Table 1:** Variation in the number of folds of falx cerebelli

Sl. No	No. of folds of falx cerebelli	Cadevers in % (*n* = 48)
1.	1 Folded	87.5% (*n* = 42)
2.	2 Folded	8.3% (*n* = 4)
3.	Multi folded	4.2% (*n* = 2)

### Single folded falx cerebelli

**Fig. 2:**
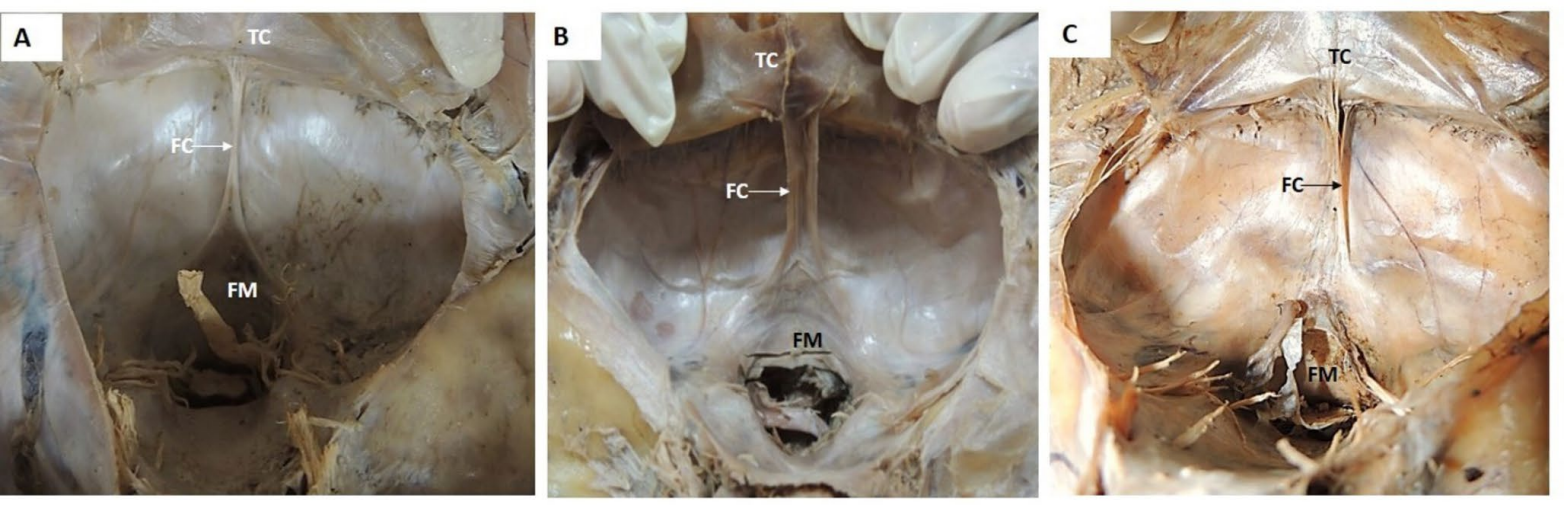
Transverse section of cranial cavity showing single folded falx cerebelli: **2A**. Single falx cerebelli; **2B.** Single falx cerebelli with broad free margin, **2C**. Indistinct falx cerebelli. FC- Falx cerebelli; TC- tentorium cerebelli; FM – foramen magnum with the brainstem

The variations were observed in the morphometry of single folded falx cerebelli. Out of 42 specimens of the single folded falx cerebelli, 33 specimens showed thin, sharp free margins (Fig. 2A), eight specimens showed broad free margins with a shallow depression (Fig. 2B), and one specimen of falx cerebelli was short and prominent in the upper part, and as it approached the foramen magnum, it became diminished, indistinct, and merged with the dura mater of the foramen magnum (Fig. 2C).

### Double folded falx cerebelli

**Fig. 3:**
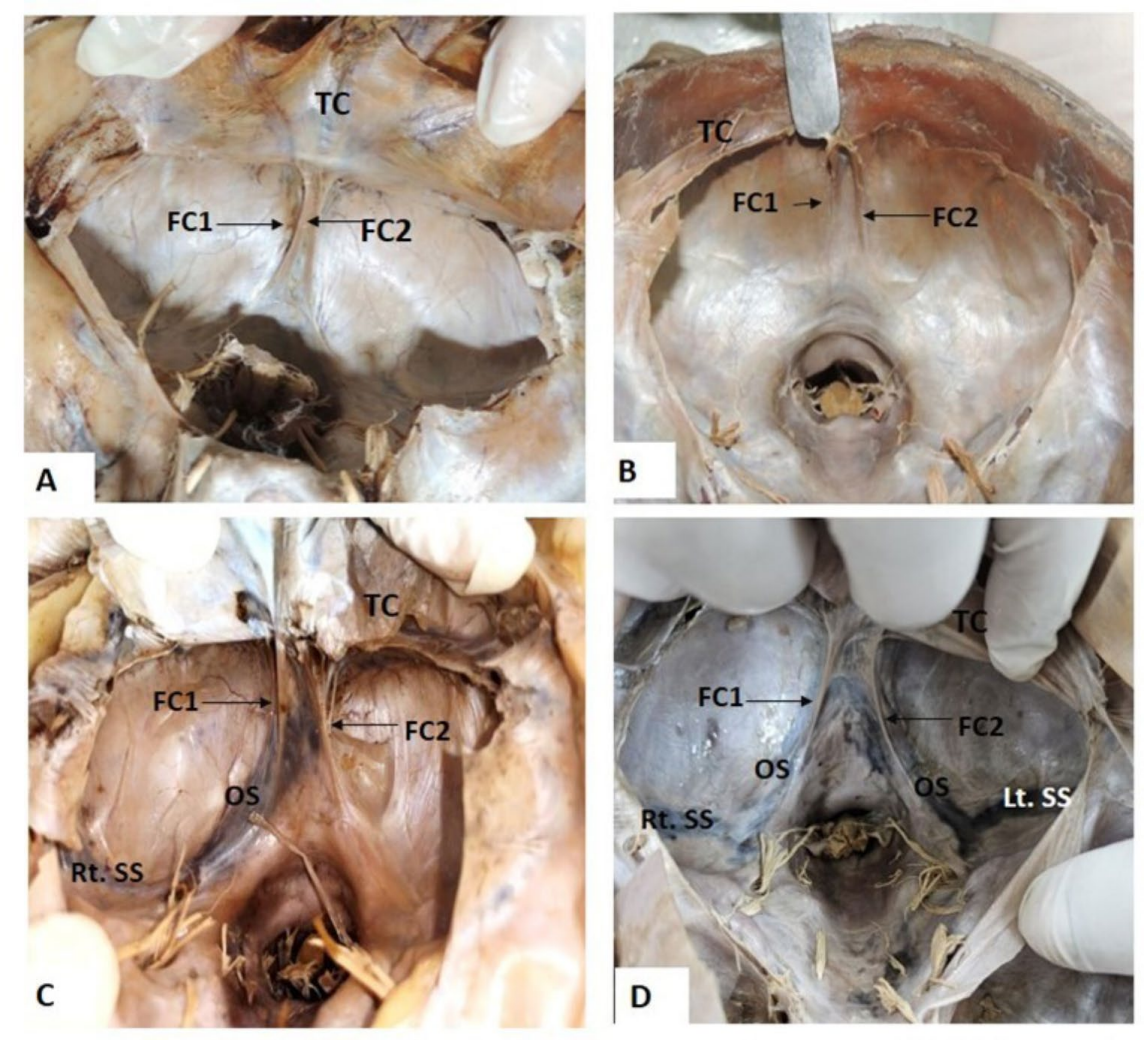
Transverse section of cranial cavity showing double folded falx cerebelli: **3A** Double falx cerebelli; **3B** Indistinct double falx cerebelli; 3**C** Double falx cerebelli with single prominent occipital sinus draining into the right sigmoid sinus; **3D** Double falx cerebelli with prominent occipital sinus draining into the right and left sigmoid sinus respectively. FC-Falx cerebelli; TC-tentorium cerebelli; FM –foramen magnum with brain stem; FC1 and FC2 are the folds of the falx cerebelli; OS-Occipital sinus; Rt SS-Right sigmoid sinus, Lt SS-Left sigmoid sinus

Double-folded falx cerebelli were seen in four specimens. In the first two specimens, both the folds of the falx cerebelli were completely extended from the lower surface of the tentorium cerebelli to the foramen magnum. The two folds were identical to each other in size, shape, and extent (Fig. 3A). In the third specimen, two folds of falx cerebelli were not identical; the fold to the right was comparatively longer than the fold to the left, however, both folds showed a diminished appearance as they approached the foramen magnum (Fig. 3B). In yet another specimen, two folds of falx cerebelli were seen, with a wide space in between them. Furthermore, a prominent occipital sinus was observed. However, this occipital sinus was seen to connect the confluence of the sinus and the right sigmoid sinus (Fig. 3C). In the last specimen of this category of double-folded falx cerebelli, a prominent occipital sinus was observed in each fold of falx that was connecting the confluence of the sinus to the right and left sigmoid sinus (Fig. 3D).

### Multi-fold falx cerebelli

**Fig. 4:**
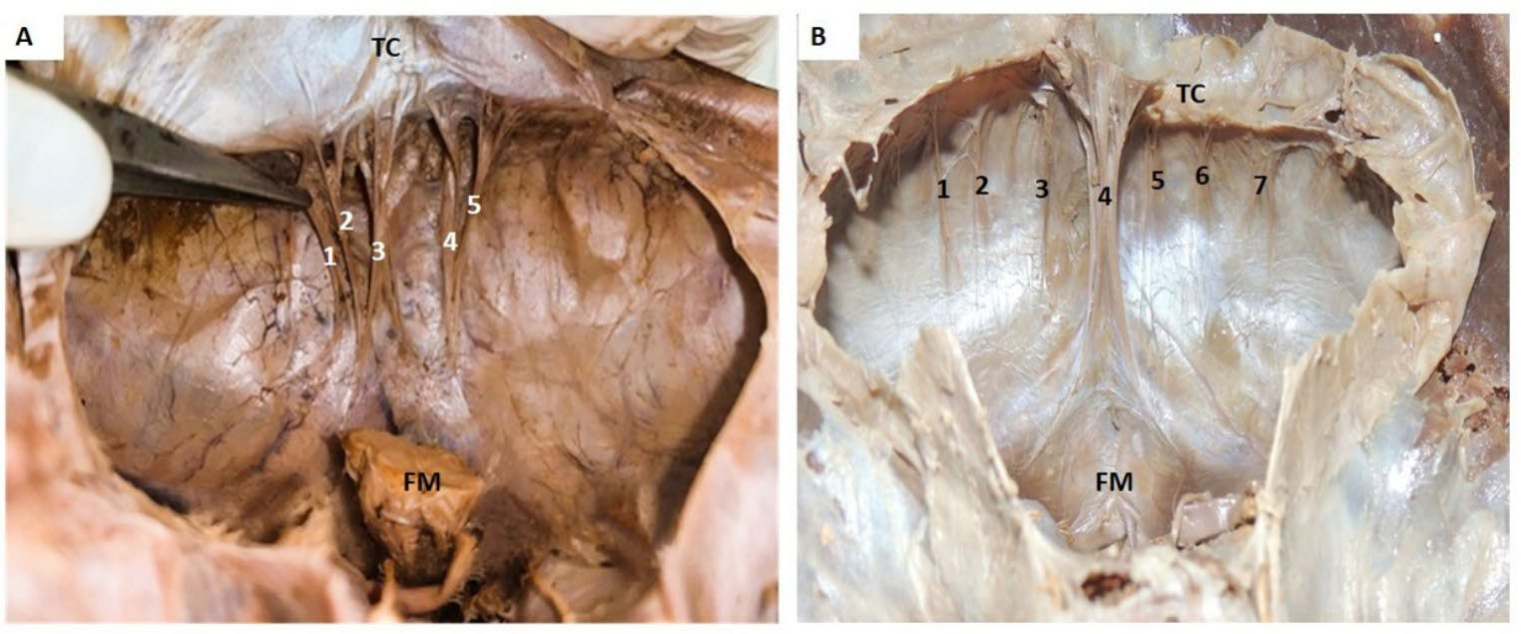
Transverse section of cranial cavity showing multi-folded falx cerebelli: **4A** Showing 5 folded falx cerebelli and **4B** Showing 7 folded Falx cerebelli.   TC-tentorium cerebelli; FM–foramen magnum with brainstem; 1,2,3,4,5,6,7 are the folds of the falx cerebelli

In two specimens, multi-folded falx cerebelli was observed. In the first specimen in this category, multiple small distinct five folds were observed instead of a single fold of falx cerebelli. Five folds were of different heights, prominent only in the upper part, separated by distinct gaps. All the folds were continuous above the tentorium cerebelli. Distally these merged with the dura mater of the posterior cranial fossa at various distances from the foramen magnum (Fig. 4A). In yet another specimen of seven folds, a single median prominent fold of falx cerebelli was observed in the center. Three more folds were identified bilaterally on either side of the median fold. The peripheral folds were shorter, smaller, and thinner than the median fold. All the folds were widely spaced (Fig. 4B).

### Proximal and distal attachments of the falx cerebelli

**Fig. 5:**
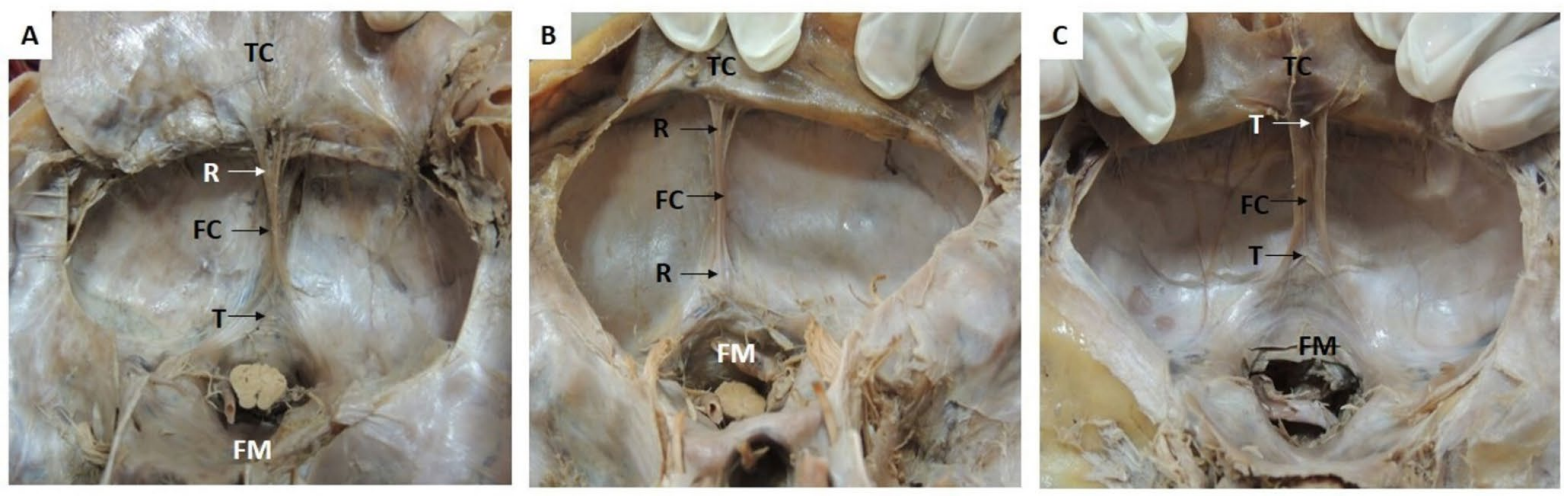
Transverse section of cranial cavity showing proximal and distal attachment of the falx cerebelli. **5A** Proximally ramified and distally triangular type of attachment; **5B** Both proximally and distal ramified types of attachment; **5C** Both proximal and distal triangular types of attachment. FC-falx cerebelli; TC-tentorium cerebelli; FM–foramen magnum with brainstem; R-ramified; T-triangular

Irrespective of the number of folds, the proximal and distal attachments of the falx cerebelli were observed, and the variations were recorded. Triangular and ramified were the two patterns observed in the attachment of the falx cerebelli. The details of the attachment patterns are shown in Fig. 5A, B, C; Table 2.

The following patterns of attachment were observed:

1. Ramified (proximal) – Triangular (distal).
2. Ramified (proximal) – Ramified (distal).
3. Triangular (proximal) – Triangular (distal).

**Table 2:** Variation in the proximal and distal attachments of the falx cerebelli

Sl. No.	Variation in the No. of folds of falx cerebelli	Variation in the attachments of falx cerebelli		Cadavers in % (*n* = 48)
		Proximal attachment	Distal attachment	
1.	1-Fold (Normal)	Ramified	Triangular	66.7% (*n* = 32)
		Ramified	Ramified	12.5% (*n* = 6)
		Triangular	Triangular	8.3% (*n* = 4)
2.	2-Fold	Ramified	Triangular	8.3% (*n* = 4)
3.	Multiple fold	Ramified	Triangular	4.2% (*n* = 2)

### Histology of falx cerebelli and the associated occipital sinus

**Fig. 6:**
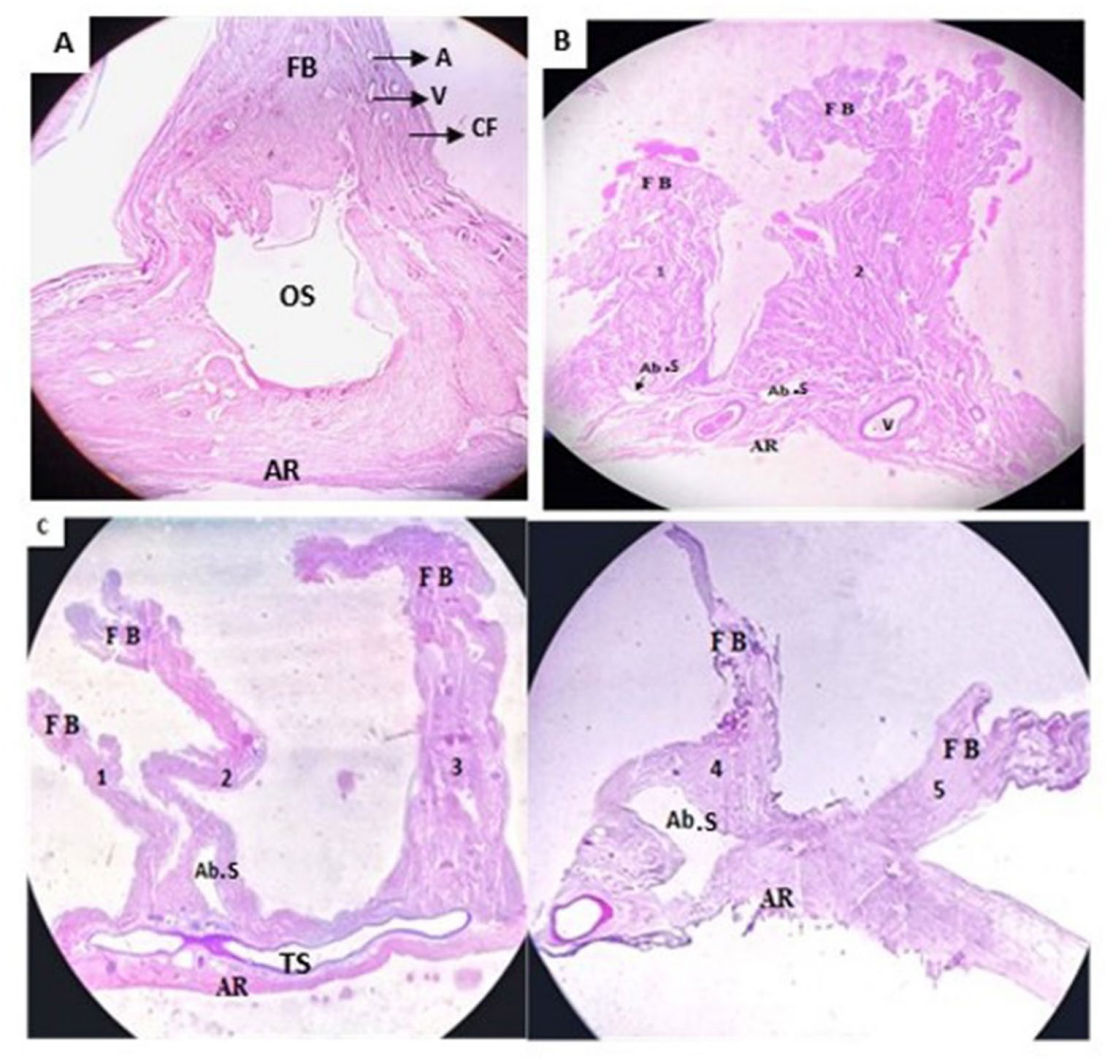
Representative images for histological sections of falx cerebelli taken from a dissection microscope. **6 A** H&E stained, transverse section of single folded FC; **6B** Double folded falx cerebelli with small indistinct aberrant venous sinus; **6 C** Five folded falx cerebelli with aberrant venous sinus in the 2nd and 4th folds of the falx cerebelli. FB-free border; AR-attached region; OS–occipital sinus; Ab.S-aberrant venous sinus; TS-Transverse sinus; V–venule; A-arteriole; CF-collagen fibers

The Haemotoxylin &Eosin(H&E) section of the single folded falx cerebelli exhibits a large, prominent, well-defined occipital sinus lined by the endothelium. Around the occipital sinus, well-defined dense irregular connective tissue comprising of collagen fibers and the fibroblast was noted. Multiple small arterioles and venules were also spotted in the connective tissue (Fig. 6A). However, the H&E section of the double-folded falx cerebelli exhibited a small, aberrant venous sinus in both folds (Fig. 6B). On the contrary, the histological section of falx cerebelli with five folds showed aberrant venous sinuses only in the 2nd and 4th folds of the falx cerebelli, while the rest of the folds showed the absence of venous sinuses (Fig. 6C).

### The drainage pattern of occipital sinus and aberrant venous sinus with relation to falx cerebelli

### Macroscopically

In 1% of the cases, the occipital sinus was seen to drain into the right sigmoid sinus (Fig. 3C). And in 2% of the cases, the duplicate occipital sinus was seen draining into the right and left sigmoid sinus (Fig. 3D).

### Microscopically

**Fig. 7:**
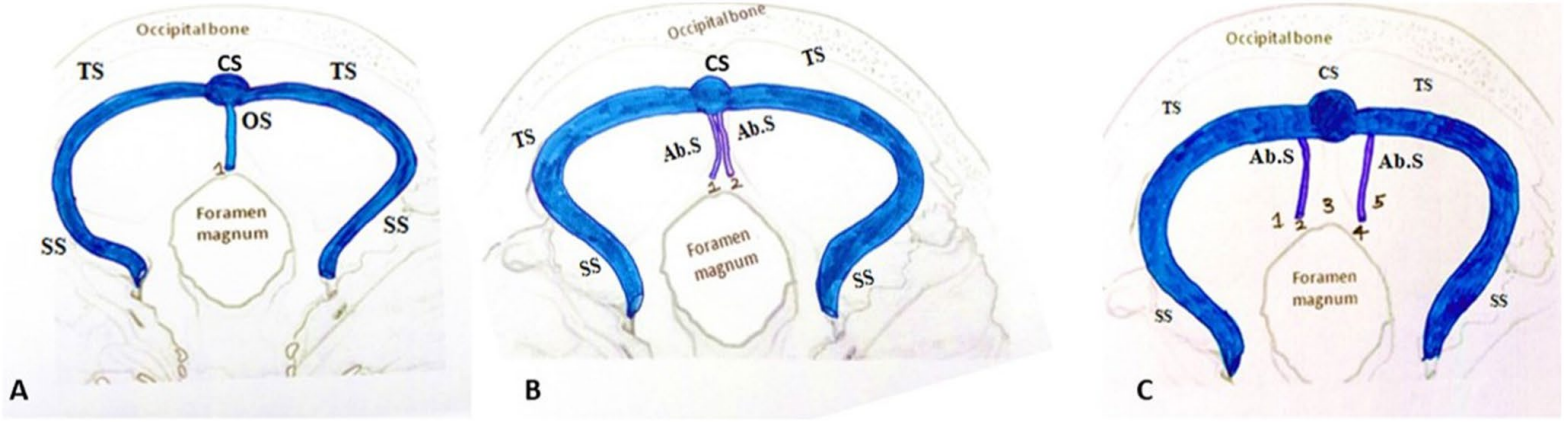
Schematic representation of drainage pattern of occipital sinus within single, double, and multiple falx folds: TS-Transverse sinus; SS-sigmoid sinus; OS-Occipital sinus, CS-confluence of sinus, Ab.S Aberrant venous sinus, 1,2,3,4,5-multiple folds of falx cerebelli

In 3% of the cases, single-folded falx cerebelli showed the presence of a single prominent occipital sinus within the falx fold which drained into the confluence sinus (Fig. 7A). In 1% of the cases, double-folded falx cerebelli showed two aberrant, small venous sinuses present in association with the falx cerebelli draining into the confluence of the sinus (Fig. 7B). In multi-folded falx cerebelli, five folded falx cerebelli showed a prominent, larger venous sinus in the 4th fold of falx cerebelli, whereas the 2nd fold had a small, aberrant venous sinus. Both the aberrant venous sinus appeared to drain into the transverse sinus. The first, third, and fifth falx folds entirely lacked sinuses (Fig. 7C).

## Discussion

The changes in the number of folds of falx cerebelli, the type of attachments (both proximal and distal) associated with venous sinus, and histological findings revealed that many types of variations are present. These variations help neuroradiologists and neurosurgeons interpret radiological imaging and plan suboccipital surgeries to avoid complications.

Variations in the presence of falx cerebelli morphometry and morphology are reported in the literature. The presence of double fold of falx cerebelli [3, 5, 9, 19] and triple fold [3,18,1] and absence [25, 26] were commonly reported in the past. Along with this, in a study done on Turkey’s population, the researchers found quadruplet (2%) and five-fold (2%) [3]. A CT scan/MRI study of children with the Chiari II malformation interestingly reported the absence of falx cerebelli and internal occipital crest. It was interpreted that the crammed posterior cranial fossa impedes the formation of folds [14].

Similarly, in our study, the variation in the number of folds of falx cerebelli was classified into single folds, double folds, and multiple folds. Single fold of falx cerebelli, which is the usual type, was seen in more than 75% of the cases. However, in the present study, we have also recorded the intricate details of the attachment of single-folded falx cerebelli, which have not been chronicled in previous studies. Further, double falx cerebelli was noted in 11% of cases. In the present study five-folded and seven-folded cerebelli, which are rarely recorded in literature, were observed. However, in the current study, three and four-folded falx cerebelli and cases without falx cerebelli were not detected in any specimens.

Previous studies noted the morphological findings of falx cerebelli of only single-folded falx cerebelli and documented its variation in the proximal and distal attachments [3]. Nevertheless, in the present study, we have noted the variation in the attachments of all folds of falx cerebelli and classified them into different types.

The combination of variation in the proximal and distal attachment of single fold of falx cerebelli in the past study was as follows - triangular and triangular type was seen in most of the specimens followed by triangular and ramified type [3]. On the contrary, in the present study, the ramified and triangular type of attachment was seen in most, and the ramified was seen in the least number in single folds; this type of attachment is unique in the current study and was not recorded in the previous study. Further, a triangular ramified type of attachment is not observed in the present study. Additionally, in our study, the variation of attachments in all the double folds and the multiple folds of falx cerebelli is opined as ramified and triangular type.

The occipital sinus is located at the attachment of the falx cerebelli. The occipital sinus usually communicates superiorly with the confluence of sinuses and inferiorly with the vertebral venous plexus at the foramen magnum [1]. However, numerous anatomical variations and studies imply that the occipital sinus is an important drainage route of intracranial veins similar to sigmoid or transverse veins [6, 13, 16]. According to reports of CT venography, the occipital sinus shows many types of variations in cases especially with a hypoplastic transverse or sigmoid sinus. Such as oblique occipital sinus extending from confluence to sigmoid sinus are seen in most of the patients with absence or small transverse sinuses [24, 29]. Also, enlarged, double occipital sinus or drainage directly into an internal jugular vein is likewise associated with hypoplastic sigmoid sinus [2]. In literature, researchers observed that in two folded falx cerebelli, a double occipital sinus independently drains into the respective right and left transverse sinuses [4]. In another similar study, it was observed that the presence of an occipital sinus was associated with two aberrant paramedian venous sinuses on either side. The two aberrant venous sinuses connected the right and left transverse sinuses into the respective sigmoid sinuses [18]. In literature, there are reports of the occipital sinus being quite large and being the main drainage canal, replacing one of the sigmoid sinuses. This is especially true in cases of an absent transverse sinus [17, 23, 27]. Triple occipital sinus is rare [15] and in some conditions, the occipital sinus is composed of a mesh of venous collaterals [22]. A single occipital sinus may be midline in position, but sometimes the position may deviate to the right or left side. Interestingly, an MRV (Magnetic resonance venography) study of 500 patients done between different ages showed no significant variation in transverse sinus morphology between genders and across ages [9]. All these studies and observations were made macroscopically in gross specimens. However, in the present study, a microscopic examination of the flax cerebelli was also conducted to avoid missing any small aberrant occipital sinus.

The histological section of falx cerebelli recorded in the literature shows the presence of fibroelastic tissue within the captured field of H&E stained tissue. Other structures seen are arterioles, venules, lymphatic vessels lined by a single layer of endothelial cells, and peripheral nerve bundles [3, 21] Similarly, our present study includes the processing of the falx fold to see not only its composition but also its association with the occipital sinus. Histological sections of single, double, and multiple folds of falx cerebelli were processed to observe the presence and drainage pattern of occipital/aberrant venous sinus. Upon microscopic observation, the results of the present study are also in consensus with the findings in the literature. In the current study, the researchers found that the single fold had one single occipital sinus, the double fold had no occipital sinus instead, it had two aberrant venous sinuses, whereas the multiple type (here five-folded) had no occipital sinus and had two small aberrant venous sinuses in the second and fourth fold of the five-fold type, whilst the rest of the folds had no venous sinuses. However, in one of the cases, we observed a large, prominent occipital sinus draining into the right sigmoid sinus, even with a normal transverse sinus.

During development, the rapid growth of the cerebrum leads to the ballooning of the transverse sinuses, in the lack of increase in the diameters of sigmoid and internal jugular veins, which leads to the formation of occipital sinus and marginal sinus [20]. Hence, the anatomical changes in the dural venous sinuses in the posterior cranial fossa are directly related to brain development.

The presence or absence of the falx cerebelli typically presents without symptoms. However, an increase in the number of folds can result in a reduction of the cranial cavity’s capacity and also affect the dural venous sinus drainage pattern. The variations in the occipital sinus are known to complicate the surgical approach of the fourth ventricular mass resection [12]. Therefore, careful attention should be paid during posterior cranial fossa surgery, especially in patients with hypoplastic transverse sinus. Comprehension of morphological alterations in dural venous sinuses is also essential to avoid misdiagnosing thrombosis. Hence, identifying alterations in the occipital sinus and dural folds becomes crucial, for planning and customising the incision to avoid sinus injury and to minimize the risks of potentially fatal complications, as they can possibly lead to internal hemorrhages. This knowledge is needed for practitioners in neurological surgery and neuroradiological image interpretation [12, 21], stressing the requirement for a nuanced approach in handling such cases. Therefore, through this study we recommend a contrast venography of dural sinus drainage pattern evaluation, to avoid risk in suboccipital craniotomy.

## Conclusion

In the existing current investigation, we explored the anatomy of the falx cerebelli, concentrating on its attachments, number of folds, and variability. Moreover, a comprehensive assessment of the microscopic anatomy of the falx cerebelli and its contents was undertaken. The findings exposed a prominent variability in both the number of folds and the pattern of attachment of the falx cerebelli.

The microstructural interpretations presented in this study impact valuable insights into the existing literature. Histological sections of the falx cerebelli offer an indication of the potential occurrence of aberrant venous sinuses, whose anomalous drainage could demonstrate challenges during posterior cranial fossa surgeries. Realizing the importance of assessing venous sinus drainage by sinus venograms before initiating any brain surgery is fundamental, as overlooking this aspect and continuing with a surgical midline incision may result in internal hemorrhage. This emphasizes the necessity of an accurate and precise understanding of the falx cerebelli’s anatomical intricacies to ensure optimal surgical outcomes.

## Data Availability

No datasets were generated or analysed during the current study.
